# The ticking time bomb in lifestyle-related diseases among women in the Gulf Cooperation Council countries; review of systematic reviews

**DOI:** 10.1186/s12889-017-4331-7

**Published:** 2017-06-02

**Authors:** Mashael K. Alshaikh, Filippos T. Filippidis, Hussain A. Al-Omar, Salman Rawaf, Azeem Majeed, Abdul-Majeed Salmasi

**Affiliations:** 1 Department of Primary Care and Public Health, School of Public Health, Faculty of Medicine, Charing Cross Campus, St Dunstan’s Road, 3rd Floor, Reynolds Building, London, W6 8RP UK; 2 Pharmacy Department, King Saud University, Medical City, Riyadh, Saudi Arabia; 3 National Heart & Lung Institute, Faculty of Medicine, Imperial College London, London, UK

**Keywords:** Cardiovascular disease, Noncommunicable diseases, Obesity, Diabetes, Hypertension, Smoking, Physical inactivity, Metabolic syndrome, Systematic review

## Abstract

**Background:**

This study aims to review all published systematic reviews on the prevalence of modifiable cardiovascular disease risk factors among women from the Gulf Cooperation Council countries (GCC). This is the first review of other systematic reviews that concentrates on lifestyle related diseases among women in GCC countries only.

**Method:**

Literature searches were carried out in three electronic databases for all published systematic reviews on the prevalence of cardiovascular disease risk factors in the GCC countries between January 2000 and February 2016.

**Results:**

Eleven systematic reviews were identified and selected for our review. Common reported risk factors for cardiovascular disease were obesity, physical inactivity, diabetes, metabolic syndrome and hypertension. In GCC countries, obesity among the female population ranges from 29 to 45.7%, which is one of the highest rates globally, and it is linked with physical inactivity, ranging from 45 to 98.7%. The prevalence of diabetes is listed as one of the top ten factors globally, and was reported with an average of 21%. Hypertension ranged from 20.9 to 53%.

**Conclusions:**

The high prevalence of lifestyle-related diseases among women population in GCC is a ticking time bomb and is reaching alarming levels, and require a fundamental social and political changes. These findings highlight the need for comprehensive work among the GCC to strengthen the regulatory framework to decrease and control the prevalence of these factors.

## Background

Cardiovascular diseases (CVD’s) remain the leading cause of death worldwide [1], resulting in more than 17.9 million mortalities in 2015. More than 3 million of such deaths occurred in people under the age of 60, which could have been largely prevented [1, 2]. The World Health Organization (WHO) and other organizations such as the American Heart Association (AHA) have recognized many risk factors, some of which are modifiable. These include hypertension (HTN), diabetes, obesity and metabolic syndrome (MetS) [3, 4]. In addition, many unhealthy lifestyles like smoking, physical inactivity, high consumption of carbohydrates and fatty foods have been identified as factors that increase the risk of CVD [5]. Rapid economic growth as well as urbanization have been also associated with higher consumption of unhealthy foods and lower physical activity, which may increase the risk of CVD [6].

The Gulf Cooperation Council (GCC) is a political and economic alliance of six Middle Eastern countries that includes the Kingdom Saudi Arabia (KSA), Bahrain, Oman, Qatar, the United Arab Emirates, (UAE) and Kuwait. The GCC was established in 1981 to ensure mutual investment and free trade between its member countries. This agreement also contributed to improvements in several fields including: education, culture, tourism, social opportunities, and health among member states [7]. Life in the GCC has changed dramatically after the discovery of oil, which became the main revenue for financing healthcare services. However, the recent fluctuation in the price of oil has affected the healthcare budget. Although GCC countries are examining different options to finance the healthcare service, up to this point, there is no clear alternative or implemented approach to achieve this goal [8, 9]. In 2013, Chahine et al., calculated the direct and indirect costs of five selected non-communicable diseases (NCD) in the GCC was $36.2 billion, where specifically, the cost of CVD and diabetes reached over $11 billion. This cost is estimated to increase to $67.9 billion by 2022, which is equivalent to one and a half times the healthcare budget of the six governments (see Table 1: The direct and indirect factors of the five selected NCDs in the GCC) [10]. However, with these healthcare expenses, the current healthcare systems adopted by some of the GCC countries is below what is available in middle-income countries [9].

**Table 1:** The direct and indirect of the five selected NCD in the GCC in 2013 [10]

	Direct cost %	Direct cost	Indirect cost %	Indirect cost	Both Direct & indirect
Condition		6,000,000,000		31,000,000,000	Total $37 Billion
Diabetes Mellitus	26	1,560,000,000	2	620,000,000	6
Cardiovascular	28	1,680,000,000	25	7,750,000,000	25
Respiratory	17	1,020,000,000	11	3,410,000,000	12
Neuropsychiatric	18	1,080,000,000	22	6,820,000,000	21
Malignant neoplasms	11	660,000,000	40	12,400,000,000	35

The prevalence of CVD risk factors, especially physical inactivity and obesity, is particularly high among women in the region [11]. This is highlighted by a report published by the Gulf Registry of Acute Coronary Events, which found that among 7900 patients with acute coronary syndrome, women had significantly higher prevalence of HTN, diabetes, and hyperlipidemia compared to men. Women were also diagnosed with unstable angina and non-ST-segment elevation myocardial infarction more frequently than men [12, 13]. Beside, women at higher risk especially in third world countries due to less access to health service, and use of medications [14]. In addition, growing evidence shows that gender inequality in income, education, health care, nutrition and political voice are strongly associated with poor health and well-being [15], making these issues extremely relevant to Arab countries in general and GCC in particular, where gender inequality is substantial [11, 16]. Such inequalities are reflected in the literature; studies focusing on women in GCC countries are limited, despite the magnitude of the problem. This review aims to provide a comprehensive overview of the modifiable CVD risk factors among women in GCC in order to inform clinicians and decision-makers in the region.

## Methods

**Fig. 1:**
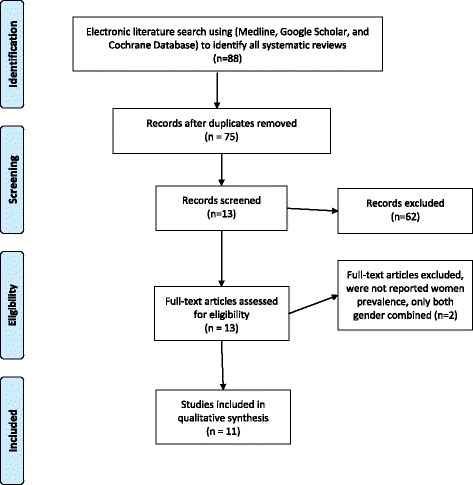
Flow Chart of the Selected Studies

Electronic literature searches for all systematic reviews published from January 2000 to February 2016 were conducted to identify all systematic reviews of CVD risk among women in the GCC region. The search was carried out in the following electronic databases: Medline, Google Scholar, and Cochrane Database (see Table 2 for search terms). No language restrictions were applied. Throughout this review, special attention was given to the modifiable risks such as HTN, diabetes, obesity, MetS, physical inactivity and smoking. Unhealthy diet, although a known CVD risk factor, was not explored in this study. The effect of diet on health is complex and different studies have focused on either overall diet patterns or individual components that include salt, sugar, fat content, fruit and vegetables, also, Also the problem with an acceptable definition of healthy diet. Hence, a comprehensive assessment of unhealthy diet would warrant a separate review. We included all systematic reviews that reported the prevalence of CVD risk factors among women in the GCC region countries. We excluded studies that reported combined data for both genders without separate prevalence for women. However, all included studies that reported the differences between genders were documented to compare gender differences in the prevalence of CVD risk factors. Any other systematic reviews from the Middle East and North Africa that included any individual GCC countries were also included. Abstracts of reviews were inspected by two authors (MA, HA) and those appearing to meet the inclusion criteria were retrieved and read in full by both authors (see Fig. 1). The quality of those studies was assessed by two authors using the Assessment of Multiple Systematic Review Tool (AMSTAR), a tool which has been validated as a means to assess the methodological quality of systematic reviews [17]. It uses an 11 point scale, where the maximum score is 11. Scores 0–4 indicate low quality, 5–8 moderate quality, and 9–11 high quality [18]. The data has been extracted independently by two researchers (MA, HA). Any disagreements were resolved by discussion between them (See Table 3: Quality assessment for reviewing the systematic reviews (AMSTAR®).

**Table 2:** selected search terms

Cardiovascular disease	
(1) “Cardiovascular disease” OR “Epidemiology of cardiovascular disease” OR “Coronary heart disease” OR “epidemiology of coronary heart disease” OR “Vascular Diseases”	
CVD risk factors	
(2) “Cardiovascular risk factor” OR “coronary heart disease risk factor” OR “stroke risk factors” OR “diabetes mellitus” OR “epidemiology of diabetes mellitus” OR “NIDDM” OR “dyslipidemia” OR “epidemiology of dyslipidemia” OR “hypercholesterolemia” OR “high cholesterol” OR “smoking” OR “tobacco use” OR “Hookah Smoking” OR “Waterpipe Smoking” OR “epidemiology of smoking” OR “hypertension” OR “high blood pressure” OR “epidemiology of hypertension” OR “obesity” OR “overweight” OR “BMI” OR “epidemiology of obesity” OR “physical activity” OR “exercise” OR “epidemiology of physical activity” OR “Metabolic Syndrome X” OR “Metabolic syndrome”	
The Gulf region	
(3) “Gulf region” OR “Arab countries” OR” GCC” OR “Middle east” OR “Arabs” OR “Saudi Arabia” OR “Kuwait” OR “Oman” OR “Bahrain” OR “Qatar” OR “United Arab Emirates” OR “UAE”	
Review	
(4) “Review, Multicase” OR “Review Literature” OR “Review, Academic” OR “Review, Systematic” (5) #1 AND #3 (6) #2 AND #3 (7) #3 AND #4 (8) #1 AND #3 AND #4 (9) #2 AND #3 AND #4

**Table 3:** Quality assessment for reviewing the systematic reviews (AMSTAR®)

	(Aljefree & Ahmed, 2015) [26]	(Alharbi et al., 2014) [21]	(Alhyas et al., 2012) [24]	Musaiger and Al-Hazzaa 2012 [23]	Alhyas et al., 2011) [25]	(S. W. Ng et al., 2011) [28]	(Musaiger, 2011) [22]	(Akl et al., 2011) [58]	(Mabry et al., 2010a) [38]	(Mabry et al., 2010b) [66]	(Motlagh et al., 2009) [27]
1. Was an ‘a priori’ design provided?	No	No	Yes	Yes	Yes	No	No	Yes	No	No	Yes
2. Was there duplicate study selection and data extraction?	Yes	Yes	Yes	No	Yes	No	No	Yes	Can’t answer	No	No
3. Was a comprehensive literature search performed?	Yes	Yes	Yes	Yes	Yes	Yes	Yes	No	Yes	Yes	No
4. Was the status of publication (i.e. grey literature) used as an inclusion criterion?	No	No	Yes	No	No	Yes	No	No	No	No	No
5. Was a list of studies (included and excluded) provided?	Yes	No	Yes	Yes	Yes	Yes	Yes	Yes	Yes	No	No
6. Were the characteristics of the included studies provided?	Yes	Yes	Yes	No	Yes	Yes	No	Yes	Yes	Yes	Yes
7. Was the scientific quality of the included studies assessed and documented?	Yes	No	Yes	No	Yes	No	No	Yes	Yes	Yes	No
8. Was the scientific quality of the included studies used appropriately in formulating conclusions?	No	No	Yes	No	Yes	No	No	Yes	No	Yes	Yes
9. Were the methods used to combine the findings of studies appropriate?	No	No	No	n/a	n/a	n/a	n/a	n/a	n/a	n/a	Yes
10. Was the likelihood of publication bias assessed?	No	No	No	n/a	n/a	n/a	n/a	n/a	n/a	n/a	n/a
11. Was the conflict of interest included?	Yes	Yes	Yes	No	Yes	Yes	No	Yes	Yes	Yes	Yes
Total/11	6	4	9	3	8	5	2	7	5	5	5

## Results

Thirteen out of 88 systematic reviews were deemed to meet inclusion criteria; however, two of them were excluded as they report results for both genders combined [19, 20]. As a result, only 11 of them were considered in this paper (See Figure 1). The majority of these studies are conducted in Saudi Arabia (Table 4). The quality of most of them was moderate according to the AMSTAR criteria [18]. Three studies were identified as low quality [21–23] and one as high [24]. (See Table 3 for more information).

**Table 4:** Data Extraction

#	Author/ Year of publication	Search engine/yrs.	CVD Risks Included in the Study	Definition	Results* CVD risk factors among women in GCC Countries	Limitation & comments	Gender/Age prevalence & Summary
1	(Aljefree & Ahmed, 2015) [25]	ProQuest Public Health, MEDLINE, PubMed, Google Scholar, and World Health Organization (WHO) website, from 1990 and 2014	• Obesity • DM	All used WHO in defining the obesity. WHO (BMI >30 kg/m2) All but one study used the WHO definition to describe DM. In UAE define DM fasting blood sugar > = 7 mmol/l or on medication All but one study uses WHO definition to report HTN Bahrain HTN > =160/95 or on HTN medications KSA, UAE current smoking definition (1 cigarette per day)	all but one study used from national / regional studies on GP, one on SP Obesity Qatar (1) study = 33.6% Kuwait (2) studies = 43% & 53% Oman (2) studies =23.8% & 26.1% KSA (4) studies = (26.6%,34%,44%,) 51.8% UAE (3) studies .2 studies = (38.3%, 35%), 1 study among SP = (6.7%) Bahrain (2) studies = (33.2%–48.7%) DM Qatar (1) study = 18.1% Kuwait (2) studies = (14.8%, 6%) Oman (3) studies = (11.3%, 11.9%, 12.1%) KSA (3) Studies = (21.5%, 20% & 44%) UAE (2) studies = (17.9% & 19.2%) Bahrain (2) Studies. 1st study report according to age group Age 50–59 = (36%) Age 60–69 = (37%) 2nd study = (5%) Hypertension Qatar (2) studies = (31.7%- 33.6%) Oman (3) studies = (22.7%, 13.8%, 31%) KSA (2) Studies = (23.9%–29%) UAE (2) studies = (20.9%–53%) Bahrain(1) Study reported according to the age group: In 50–59 age = (33%) In 60–69 age = (43%) Smoking Qatar (1) study = (3.2%) Kuwait (2) Studies. 1st Study reported both type of smoking among SP. Cigarette = (7.9%) Water-pipe = (5.5%). 2nd study overall smoking = (1.9%) Oman (2) studies = (0.5%–0%) KSA (2) studies = (1–9%) UAE (1) study = (0.8%) Bahrain (1) study. Cigarette smoking = (3.2%), Water-pipe = (17.5%) total = (20.7%) Physical Inactivity Kuwait (1) study = (80.8%) Oman (1) study = (69.3%) KSA (1) Study = (98.1%) UAE (1) study = (56.7%) not walking daily 20 min. Bahrain (1) study.t report Walk 1–3 km = (6%) Walk less than 1 km = (93%)	Lack of recent nationally representative reports in the GCC countries, and thus it is difficult to compare the data between GCC countries. There was significant heterogeneity between studies with respect to definitions of the risk factors, design and population characteristic. Few studies focusing on HTN, dyslipidaemia and physical activity. Studies relating to the prevalence of risk factors in Qatar and Bahrain were also relatively low. Most studies cited were publish before 2000.	Gender/Age & obesity • The prevalence of obesity in males ranged from 10.5% to 39.2% and in females ranged from18.2% to 53%. Higher in female than male. • The prevalence of obesity increased with age with the highest level in the middle age groups (30–39 and 40–49 years). Gender/Age & DM • Three studies showed higher DM rates among females, while three studies indicated the opposite. Four studies showed almost no difference in the prevalence of diabetes between genders • The prevalence of diabetes rose proportionally with age and reached the highest rates in both sexes among those aged 55–64 years and over. Gender/Age & HTN • Rate of HTN in GCC states ranged from 26% to 50.7% in males and from 20.9% to 31.7% in females. • Across all studies, the prevalence of HTN considerably increased with age with the highest rates in the 45–65 age groups. Gender/Age & smoking • The rates of cigarette smoking in the GCC ranged from 13.4% to 37.4% in males and from 0.5% to 20.7% in females. • In females, the highest rates of smoking were in the older age group (40–49 years) Gender/Age & physical inactivity • Across all age groups physical inactivity was higher in females than males. The rates of inactivity ranged from 24.3% to 93.9% in males and from 50% to 98.1% in females in the GCC. Summary: Effective preventative strategies and education programs are crucial in the Gulf region to reduce the risk of CVD mortality and morbidity in the coming years.
2	(Alharbi et al., 2014) [20]	Medline and Embase. From 1st January 1979 to 31st December 2011	• DM • Obesity	Obesity and DM have been reported according to WHO criteria.	DM Kuwait (3) studies. Only one study reporting both type 1, type 2 DM on GP = 11.7%. 2 Studies type 2 DM only. First in PC = 8%. and the second on GP = 14.8% UAE (3) studies. (2) studies on GP = 6%,11.1% (1) Study not gender specific. Oman (3) studies. 2 Studies on GP = 10.1%–11.3% for type 2 DM only. 1 study in both type 1 + 2 = 11.3% Bahrain (1) study GP in both type 1, 2 DM =13.4%. Qatar (1) study GP in both types 1, 2 diabetic =18.1%. KSA (10) studies. 7 Studies reporting both type 1, and 2 DM from which 6 studies on GP = (13.8%, 13.8%, 17.1%, 17%, 18.3%, 21.5%). & 1 study on PC = 29.2%. 3 Studies reporting type 2 DM only from which 2 Studies on GP = (11.9% 12.2%) & 1 Study in PC = 30.9%. Obesity Kuwait (3) studies. 2 Studies in PC = (32.2%–40.6%), 1 study on GP = 34.9%. UAE (2) studies on GP = (27.5%-16%) Bahrain (2) studies on GP = (31.4% -53.2%) KSA (6) studies. 2 Studies in PC = (43.9%, 40.5%) 1 study among SP = 20.8% (3) Studies on GP = (24%, 55.2%, 49.2%). Oman (1) Study on GP =4 9.5%	The majority of the studies reviewed did not distinguish between type 1 and type 2 DM, and the studies reviewed displayed heterogeneity of methods, sample size, and age range. Insufficient data on the prevalence of obesity in adults to observe a clear trend occurring over time. Most studies cited published before 2000	Gender & DM Despite the rise in the prevalence of diabetes among Saudi women and men between 1980 and 2012 however, the trend more with men than women. They also address the need of urgent intervention such as the implementation of prevention, health promotion, and improved DM management systems. Summary: Diabetes and obesity have a higher prevalence in GCC. Among the Saudi population, the prevalence of diabetes increased from 10.6% in 1989 to 32.1% in 2009.
3	(Alhyas et al., 2012) [23]	Medline and Embase from 1982 and 2009.	• DM	KSA (total 11 study only 6 reported the gender) national	All studies have been conducted on GP sample size ≥1000 DM KSA (6) Studies = (5.9%, 3.6%, 11.8%, 9.8%, 4.53%, 21.5%) UAE (4) studies. 3 studies =2.58%,22.1% 22.3%, 1 study by age Age 20–29 = (1.7%) Age 30–39 = (5.3%) Age 40–49 = (26.2%) Age 50–59 = ((27.1%) > 60 yr. = (43.3%) Bahrain(1) study Age 50–59 = (35.4%) Age 60–69 = (37.6%) Oman (3) Studies = (10%, 11.3% 11.3%) Qatar (1) Study = (18.1%)	The major limitation of this studies was heterogeneity of the reviewed studies, and variable availability of sub group data. Most studies cited published before 2000.	Gender/Age & DM • Five studies included studies were in favor of a male. However, in nine further studies, higher prevalence, of undetermined significance (or close to significance was observed in females. A further three studies showed no significant gender difference. • Most of the studies demonstrated a significant association between advancing age and prevalence of DM Summary: The prevalence of DM is an increasing problem for all GCC states. They may therefore benefit to a relatively high degree from co-ordinated implementation of broadly consistent management strategies.
4	Musaiger and Al-Hazzaa 2012 [22]	PubMed and Google Scholar databases / between January 1, 1990 and September 15, 2011 was /102	• DM • HTN • High TC • Smoking • Physical inactivity • Obesity/ Overweight/ • MetS	WHO definitions HTN (BP ≥140/90 mmgHg TC: 5.2 mmol/dl; % Physical inactivity define as participating in PA ≤ 10 min.	All the studies has been conducted on GP DM Kuwait (1) study = 14.8% KSA (1) study = 21.7% Oman (1) study = 12.3% Qatar (1) study = 11% HTN Kuwait (1) study =19.7% KSA (1) study = 18.5% High TC Kuwait (1) study =37.2% KSA (1) study =19.7% Smoking Kuwait (1) study = 3.0% KSA (1) study = 1.2% Physical inactivity Kuwait (1) study = 71.7% KSA (1) study = 74.3% Overweight KSA (1) study = 28.8% Oman (1) study = 27.2% Kuwait (1) study = 28.9% Bahrain (1) study = 31.1% Obesity KSA (1) study = 50.4% Oman (1) study = 22.3% Kuwait (1) study = 53% Bahrain (1) study = 40.3%	No limitation subhead was provided in this review. Only national data used in this review. No standardized tools in reporting the results which makes it difficult to establish accurate results.	Gender/Age & DM • In general, the prevalence rates in men and women were very close • Age-standardized adjusted estimates for raised blood glucose in the EMR countries showed the highest prevalence among Saudi men and women (20 years and older) at 22% and 21.7%, respectively. Gender/Age & obesity • Women in the GCC were more obese than men. • Obesity was found high even among the children. Gender/Age & MetS • The prevalence MetS in the GCC was some 10%–15% higher than in most developing countries, with a higher prevalence among women. The proportion of metabolic syndrome in the GCC ranged from 20.7% to 37.2% (ATP III) definition, and from 29.6% to 36.2% using (IDF) definition. Summary: Several risk factors may be contributing to the high prevalence of N-NCDs in EMR, including nutrition transition, low intake of fruit and vegetables, demographic. Transition, urbanization, physical inactivity, hypertension, tobacco smoking, stunting of growth of preschool children, and lack of nutrition and health awareness. Many EMR countries have been reporting the onset of DM in increasingly younger age groups. Intervention programs to prevent and control N-NCDs are urgently needed, with special focus on promotion of healthy eating and physical activity.
5	Alhyas et al., 2011) [24]	Medline and Embase from 1950 to July week 1 2010, and 1947 to July 2010	• Obesity/ Overweight • DM • HTN • High TC	Overweight (if not25 to < 30) Obesity (if not ≥30)	Obesity Kuwait (3) Studies. 2 Studies in PC 40.6%, 29.9% & 1 study WP = 32.2%, KSA (6) studies. 3 studies in PC = 49.15%, 47.0%, 40.5% 3 Studies on GP =23.6%, 26.6%, 23.97% Bahrain (2) Studies from GP =31%–33.2% UAE (4) Studies. 2 Studies GP = 16%,40% 1 Study SP 9.8%, & 1 study PC: 46.5% Overweight KSA (6) Studies. 3 Studies on GP = 28.4%, 29.4%, 29.09% 3 Studies in PC =31.55%, 26.8%, 31.5% Kuwait (3) Studies. 2 Studies in PC (59.2%, 72.9%) and 1 study on WP: 32.8%. Bahrain (2) Studies on GP = (29.4%–32.7%) UAE (2) studies on GP = (27%, 35%)	Heterogeneity of the reviewed studies. Make only crude observation, and could not provide measures of confidence in the outcomes. The quality of reporting of results is also variable. Most studies cited were publish before 2000. They include School student population in their study in the same table with adult population. This study concentrated mainly on obesity and DM. The rest of CVD risk factors such as HTN and Hyperlipidaemia and their result have not been included as they reported the prevalence in both genders.	Gender/Age & obesity/overweight • prevalence of obesity and overweight was higher in women in most of the studies, and 1 study where overweight was higher in men, indeed, the combined prevalence of overweight/ obesity remained higher in women • Age as a potential predictor of prevalence of over- weight/obesity was considered in eight studies (of adult populations) specially from age,36 and a significantly higher mean BMI in a 45–54-year age group versus a 55–64-year age group Summary: There is high prevalence of risk factors for diabetes and diabetic complications in the GCC region, indicative that their current management is suboptimal. Enhanced management will be critical if escalation of diabetes-related problems is to be averted as industrialization, urbanization and changing population demographics continue.
6	(S. W. Ng et al., 2011) [28]	Medline database, PubMed Central, Academic OneFile, LexisNexis ® Academic, Google Scholar, WHO InfoBase and manual cross references from retrieved articles. English language between 1st January 1990 and 31st June 2009	• Obesity/overweight • HTN • DM	WHO definition was used: overweight (25 BMI < 30) obese (BMI 30)	All studies have been conducted on GP sample size ≥1000 Overweight/ obesity Oman (1) Study = (23.8%)/ (27.3%) Bahrain (1) Study = (28.3%)/ (34.1%) UAE Only obesity was reported (1) Study = (39.9%) KSA (1) Study = (27.6%/ 43.8%) Qatar (1) Study = (33%/45.3%) Kuwait (1) Study = (29.5%/47.9%) HTN * self-report. ** Measured HTN UAE (2) Studies = (7.8%, 11.2%)* Measured HTN = (32.4%)** Saudi Arabia (1) Study. Age 30–70 yrs. = (33.5%)** Bahrain (1) study Age 40–59 yr. s = (37.4%)** Qatar (1) Study Age 25–65 = (31.7%)** Oman (3) Studies = (6.1%)*, (26.3%)**, (31.1%)** DM * self-report. ** Measured DM UAE (3) Studies = (5.2%)*, (12.1%)* (53.1%)* KSA (2) Studies = (20%) **, (17.2%)* Bahrain (1) Study = (36.4%)** Oman (3) Studies = (9.7%) **, (11.8%) **, (3.3%)*	The only limitation that reported was the comparison of the prevalence trend for children and adolescents which is difficult due to differing standards used.	Gender/Age & Obesity/Overweight • Gender differential in the prevalence of overweight and obesity, with women having notably higher rates than men, particularly starting from their mid-20s. • Obesity is common among women; while men have an equal or higher overweight prevalence. • Among adults, overweight plus obesity rates are especially high in Kuwait, Qatar and Saudi Arabia, and especially among 30–60 year olds Gender/Age & HTN • The prevalence of HTN rose with age for all cohorts across all the countries with nationally representative data broken down by age groups. Gender/Age & MetS • Certain populations, such as Saudis, older Qataris and women in general appear have particularly high rates of MetS. Summary: • The UAE and Saudi Arabia have some of the highest prevalence and growth of hypertension. • In the UAE, prevalence of self-reported DM more than doubled between 1995 and 2000. Similarly, seen in Saudi Arabia and Oman but wasn’t sharp increase. • There is a need for continued surveillance of overweight, obesity (by various grades, not just BMI >30) and N-NCDs, particularly from nationally representative samples using clinical measures over self-report. N-NCDs are largely preventable.
7	(Musaiger, 2011) [21]	Published in English between January 1990 and May 2011 using Medline database, PubMed Center, Google Scholar, and WHO Info Base was carried out. Health ministry and other official reports which included the prevalence of overweight and obesity among preschool children, school-aged children, adolescents, and adults were also covered.	• Obesity/ Overweight	They include national big sample size studies All adult included studies used WHO definition of obesity	All the studies have been Studies conducted on GP. Obesity Bahrain (1) Study = (40.3%) Kuwait (1) Study = (53.0%) Oman (1) Study = (22.3%) KSA (1) Study = (50.4%) Overweight Bahrain (1) Study = (31.1%) Kuwait (1) Study = (28.9%) Oman (1) Study = (27.2%) KSA (1) Study = (28.8%)	No limitation subhead was provided in this review.	Gender & Overweight/ Obesity • Obesity is more prevalent among women in all countries of the EMR. The mean BMI for women is higher than that for men in all countries in the EMR. Summary: Among adults the prevalence of overweight and obesity ranged from 25% to 81.9%. Possible factors determining obesity in this region include: nutrition transition, inactivity, urbanization, marital status, a shorter duration of breastfeeding, frequent snacking, skipping breakfast, a high intake of sugary beverages, an increase in the incidence of eating outside the home, long periods of time spent viewing television, massive marketing promotion of high fat foods, stunting, perceived body image, cultural elements and food subsidize policy. In all high and middle income countries in the EMR, overweight and obesity has become a major public health problem, with a prevalence higher than many of developed countries. This creates the need for urgent action to prevent and control obesity in EMR countries. A national plan of action to overcome obesity is urgently needed to reduce the economic and health burden of obesity in this region.
8	(Akl et al., 2011) [58]	Electronically searched the following databases in June 2008, MEDLINE (1950 onwards), EMBASE (1980 onwards), and ISI the Web of Science using no language restrictions.	• Water pipe smoking	They reported smoking & whoever tried to smoke a water pipe even if once.	Water pipe Smoking Kuwait (2) Studies = (3%, 1.9%) Bahrain (1) Study = (3%) KSA (1) Study WP = (11%) UAE (1) Study = (3%)	Only four studies were conducted at national level Variation in reporting the prevalence and type of smoking. Only one study used validated tools to measure exposure to water pipe smoking. All studies included were cross sectional in design and did not allow analyses for time trends.	Gender/Age & water pipe smoking • Inconclusive evidence among genders. • No age different was reported. Summary: While very few national surveys have been conducted, the prevalence of water pipe smoking appears to be alarmingly high among school students and university students in Middle Eastern countries and among groups of Middle Eastern descent in Western countries.
9	(Mabry et al., 2010b) [66]	PubMed and CINAHL from 2003–2009 studies	• MetS	Definitions Third Adult Treatment Panel (ATPIII) of the National Cholesterol Education Program (NCEP-ATPII) and the international DM Federation (IDF) definitions are used Sitting national GP and from PC Only study reports from WHO	All the studies have been Studies conducted on GP. MetS KSA (1) Study on GP = ATPIII (42%). Qatar (1) Study = ATPIII 32.1% & 37.7% (IDF) Kuwait (1) = (36.1%) IDF UAE (2) Studies = (24.2%, 42.7%) ATPIII, (45.9%) IDF Oman(1) Study = (23%)ATPIII,(40%)(IDF)	This review focuses on Studies that are published in the English language. It is possible that additional studies are available within the grey literature (such as government reports of studies carried out by each country) as well in Arabic-language publications. There was noticed variation in the methodological quality of the studies included, non-population base sample, use of un-validated measurement instruments, and varying physical activity definitions. No standardized protocols provided.	Gender/Age & MetS • Generally higher prevalence rates were reported in women. • Studies in the GCC have reported a positive association between age and the prevalence of the MetS. Summary: Significant socio-demographic associations with the MetS identified in the individual studies include: age, women, higher income, lower educational, urban residence in Saudi Arabia, and rural residence in the UAE.
10	(Mabry et al., 2010a) [38]	PubMed and CINAHL databases. The years of starting the search not reported.	• Physical activity	They include national big sample size	All the studies have been Studies conducted on GP. Physical Inactivity KSA (3) studies = (34.3%, 73.7%, 98.1%) Kuwait (1) study = (71.6%) Qatar (1) Study = (60.5%) Bahrain (2) Study = (93%, 98.7%) UAE (1) Study = (50.7%)	This review focuses on Studies that are published in the English language. It is possible that additional studies are available within the grey literature (such as government reports of studies carried out by each country) as well in Arabic-language publications. Included only the national population in the sample. Given that the percentage of non-nationals living in the GCC states varies from 27% to 80%. The prevalence of sufficient physical activity in the overall adult population (including both national and non-national residents) may differ from what has been reported. variation in the methodological quality of the studies, including non-population-based sampling, Use of un-validated measurement instruments, and varying physical activity definitions. Lack of standardized study protocols, make it difficult for cross-country comparisons	Gender/Age & physical activity • Men were significantly more active than were women • The correlation of physical activity with age was less clear. Summary: Prevalence estimates for participation in physical activity in the GCC States are considerably lower than those for many developed countries. Given the increasing prevalence of overweight and obesity and associated chronic diseases in the GCC States, and with physical inactivity being an important and modifiable risk factor, health promotion strategies should aim to increase physical activity among both men and women as a priority public health issue.
11	(Motlagh et al., 2009) [26]	MEDLINE/PubMed was conducted for articles published from January 1980 to April 2005 in the Middle East region	• DM • Obesity • HTN • Smoking	Obesity WHO (BMI >30 kg/m2) DM used WHO definition HTN (SBP ≥ 140 mmHg)	All studies have been conducted on GP sample size ≥1000 Obesity Kuwait (3) Studies = 29.9%, 30%,40.6% Oman (4) Studies ranged =17.7%–49.5% Qatar (1) study = 45.3%. KSA (6) Studies ranged = 20.3%–32.8% DM Kuwait (1) study = 21.8% Oman (2) Studies = 9.8%,11.3% KSA (5) Studies ranged = 3.6%–21.55% HTN Oman (1) Study = 18.7% Qatar (1) study =31.7% KSA (2) Studies ranged =3.2%,22.1% Smoking Bahrain (1) study = 9.2% Kuwait (2) Studies =1.4%,1.9% Oman (2) Studies = 0.5%,1.6%, Qatar (1) study = 11.6% KSA (3) Studies ranged =0.9%–1.0%	Studies included in this review varied in study design, population include definition of risk factor. Most studies cited were published before 2000. No definition for the HTN has been given. Lack of standardized definitions of dyslipidaemia limits ability to provide summary estimates for this risk factor. No difference in diabetes between gender 2 studies association between HTN and obesity. Low prevalence of Smoking was reported due to smoking being culturally unaccepted Underreporting may occur.	Gender & reported CVD risks • Smoking was more common in men than women, whereas obesity and hypertension were more common in women. Summary: Middle East region (GCC specifically) was considerably higher among women compared with the men. Although the exact cause of such sex variations is not entirely clear, it has been reported that women are less active compared with men in certain areas. Physical and cultural barriers to physical activity have been reported among women in Saudi Arabia.

*Self-reported

**Measured

*Abbreviations*: *MetS* Metabolic syndrome, *DM* Diabetes Mellitus, *EMR* Eastern Mediterranean region,*NR* Not Reported, *GCC* Gulf Cooperation Council, *KSA* Kingdom of Saudi Arabia, *UAE* United Arab Emirates, *M/F* Male/Female, *SP* Student Population, *PC* Primary clinic, *GP* General Population, *WP* Working Population, *TC* Total Cholesterol, *N-NCDs* Nutrition related non-communicable diseases

### Obesity

Six systematic reviews reported the prevalence of obesity among women in the GCC region. Most of them adopt the WHO definition for BMI, identified as an indicator for obesity (obese: BMI ≥ 30.0 kg/m^2^). The prevalence of obesity among women in the GCC is high and ranges from 29.2% up to 45.3%. The highest prevalence was among Qatari women (45.3%); the prevalence was 38.4% in KSA and 35.2% in Kuwait. The lower prevalence levels are reported in UAE (31.3%) and Oman (29.2%) [21, 22, 25–28]. While obesity has greater prevalence in women than men, being overweight is more prevalent among men within the GCC (See Table 4).

### Physical inactivity

The prevalence of physical inactivity among the female population in the GCC region is reaching an alarming level, ranging from 50.7 to 98.7%. In 2007 Al-Nozha et al., reported the rate of physical inactivity from a large national health survey in Saudi Arabia, the result was shocking, 96% in both sex, and more was among women 98.1% [29]. Bahraini women share the same high level of physical inactivity with a prevalence of up to 98.7%, including a study showing that 93% of Bahraini women walk less than 1 km daily. Furthermore, the prevalence of physical inactivity among Kuwaiti women stands between 71.6 and 80.8%. The reviews in Qatar and Oman report a prevalence from 60.5 to 69.3% respectively. UAE stands at 50.7%, however, 56.7% of the women were inactive to the extent that they were reported to have not walked for longer than 20 min a day [26, 30].

### Diabetes

The prevalence of diabetes is high within the GCC countries. Five systematic reviews have reported such a prevalence based on sample size, >500, mainly from national surveys. Most of the studies use the WHO definition for diabetes [21, 24, 26–28]. However, several studies within the reviews combined both types of diabetes (type 1 and type 2). The prevalence among women in the GCC ranges between 6 and 44%, averaging 21% [26]. Studies (before the year 2000) report low prevalence of diabetes while reviews citing more recent studies report higher prevalence rates. For example, the review by Alhyas et al. which includes relatively new data shows higher prevalence of diabetes [24]. The prevalence of diabetes in the GCC region is higher among people above 50 [24, 26]. Unlike obesity, there is no clear gender gap in diabetes (See Table 4).

### Hypertension (HTN)

Four systematic reviews reported the prevalence of HTN in women in the GCC [23, 26–28]. An additional study did not take gender into consideration [25]. HTN among Qatari women ranges from 31.7 to 33.6%, while 33–43% of women between 50 and 69 years old were hypertensive in Bahrain. Two studies within the reviews in UAE report contradictory results.

The Aljefree and Ahmed review reports a prevalence between 20.9 to 53% while Ng, Shu Wen et al., estimated the prevalence of HTN between 7.8% to 11.2%. This result was based on self-reported data, whereas HTN measured in the same region was 32.4% [28]. Similarly, blood pressure values measured among Omani women are higher compared to selfreports (31.1% vs 6.1%) [28]. Self-reported HTN underestimates the actual prevalence of HTN because of its non-symptomatic appearance. As for Saudi Arabia, Motlagh and colleagues reported that the HTN prevalence among Saudi women ranged from 3.7% to 22.1% between 1996 and 1997 [27]. More recent studies in the review conducted by Aljefree and Ahmed show a range between 23.9% and 33.5% [26]. There were a limited number of studies that reported the prevalence among the Kuwaiti population within these reviews. With regards to gender differences, several studies have revealed slightly greater prevalence of HTN in men [20, 25, 26, 31].

### Smoking

Three reviews have reported the prevalence of smoking [26, 27, 32]. It is generally lower among women than men within the GCC region. Motlagh et al., showed that women from Qatar and Bahrain have a higher prevalence of smoking than in other GCC countries at 11.6 and 9.2% respectively, while in Saudi Arabia, Oman, and Kuwait, the prevalence ranged between 0.5 and 1.6% [27]. Aljefree & Ahmed found in their review that the prevalence of smoking among women in Saudi Arabia in 2003 was 9%, while in Oman it was 0.5%, 0.8% in UAE, 7.9% in Kuwait, and the highest prevalence was in Bahrain (20.7%), which was mainly water pipe smoking [26]. Currently, though, water pipe smoking is increasing among GCC women. The majority of the GCC countries have a similar prevalence of water-pipe smoking, which is around 3% of women. Only one study states that the percentage of Saudi women smoking water pipes is 11% [32].

### Metabolic Syndrome (MetS)

The overall prevalence of MetS among women in the GCC countries is reported by Mabry et al. using the definitions of the National Cholesterol Education Program-Adult Treatment Panel III (NCEP-ATP III)^1^ and the International Diabetes Federation (IDF).^2^ Based on ATP III criteria, the prevalence of MetS in the UAE is high (42.7% ATP III), 42% ATP III among Saudi women, and an ATP III score of 32.1% in Qatari women. The lowest prevalence, however, can be found among Omani women, with 23% ATP III [33]. The prevalence in some countries has been reported using IDF criteria instead of ATP III. In UAE, it is 45.9% IDF while in Qatar it is 37.3% IDF, and the lowest is in Kuwait at 36.1% IDF according to the studies we examined. No data on prevalence of MetS among female population in Bahrain was reported.

## Discussion

Our review showed that the prevalence of major lifestylerelated risk factors for CVD is very high among women in GCC countries and seem to be increasing over the past decades.

Obesity among Arab women is highly prevalent, with the greatest increase reported in the literature among Middle Eastern countries in the six GCC countries [34]. The prevalence of obesity among women in GCC countries is higher than in countries such as Iraq, Libya, Algeria as well as European countries [35]. With regards to the marital status, married women within the GCC are more susceptible to obesity than unmarried one [35]; one of the possible reasons is that married couples are less active and tend to eat together, which may reinforce increased food intake [36]. The WHO has announced that Gulf countries have the highest prevalence of obesity, mainly among Kuwaiti, KSA, and Bahraini women [37]. The Middle East is recording the fastest increase in obesity prevalence over time, with more women than men being obese [34]. This may be attributed to multiple factors; for example the majority of households in this region, especially in Kuwait and Saudi Arabia, commonly hire housemaids which could lead to low activity and sedentary lifestyle [38]. In addition, high consumption of fast foods (high in fat and carbohydrates) combined with a sedentary lifestyle which are norms in today’s GCC have played an important role in increasing levels of obesity in recent years [39, 40]. Multiple pregnancies can also contribute to weight gain, as women may retain an average of 4.5kg after each birth [41].

Physical inactivity is a global public health problem. Around 31% of adults aged 15 and over were insufficiently active in 2008, with women being less active than men (34% vs 28%) [42]. Physical inactivity is very common in the Muslim world especially among Arabs. Based on data from 163,556 participants in 38 Muslim countries, Arab women were more likely to be physically inactive than non-Arab women (Odds Ratio=2.15, 95% CI: 2.09–2.21) [43]. Also, in a study conducted by Daryani et al, Arab immigrants in Sweden reported a higher prevalence of abdominal obesity than Swedish-born women, and a high degree of physical inactivity during leisure time, highlighting potential cultural factors [44]. Sedentary lifestyle is very common, especially among women in the Middle Eastern countries. This could be due to various reasons. In countries such as Saudi Arabia, physical education was not included in the public girl’s school curriculum until early 2013 and women are still forbidden from driving, which limits their access to fitness centers [45]. Other barriers may include the desert climate, high temperatures and frequent sand storms, which makes it difficult to exercise outdoors, the lack of social support, and the common use of cheap migrant labor for household work [46].

Diabetes is a complex disease that is linked between multiple genetic and environmental factors including diet, lifestyle, and obesity [47]. Several studies show that Arabs have a greater genetic predisposition to diabetes than Caucasians [48, 49]. In Saudi Arabia, like other GCC countries, the prevalence of consanguinity is as high as 60%, which is considered the highest rate of consanguineous marriages in the world [50, 51] and has contributed to the high prevalence of diabetes within the GCC countries [52, 53]. Additionally, the fast urbanization and increased per capita income have had negative influences on GGC lifestyle resulting in increased sedentary lifestyle, leading to obesity [54]. Obesity is a major risk factor for developing diabetes, where in many cases, more than half of the diabetic patients were found to be obese [55, 56]. From a cost perspective, Saudi Arabia spends 21% of their total health expenditure on diabetes, with other GCC countries spending between 16 and 19% [57].

The prevalence of HTN was also high among women in GCC countries. Data from the Second Gulf Registry of Acute Coronary Events (Gulf RACE-2) showed that 47.2% of the registered individuals were hypertensive, and women were more likely to have HTN than men [13, 58]. In 2014 El Bcheraoui et al., reported the prevalence of HTN from a large national health survey of more than 10,000 households throughout KSA. The overall prevalence was 15.2% of those with hypertension were found to be undiagnosed [59]. Underreporting should not be ruled out, as many of the studies included collected self-reported data [28]. Likewise, a study published in Saudi Arabia also showed that almost 40% of people affected by HTN were unaware of their disease at the time of the survey [60].

Low prevalence of smoking among women in the GCC countries could be an indication of under reporting, as smoking cigarettes traditionally is not accepted among Arab Muslim women, especially in the GCC countries [61]. In contrast, the acceptance and popularity of water pipe smoking is very common among Arabs in general, especially women [62–66]. There is also a false perception that water pipe smoking is less harmful than cigarettes [67]. Up to this point, the data shows a growing trend of women smoking water pipes in the GCC countries, but it is still less than other neighboring Arab countries [32].

### Limitations

The heterogeneity of the reviewed studies and variable availability of sub-group data was a major limitation in the review process within the GCC countries. We presented the actual reported percentage or the range of percentages in the cited studies that pertain to the prevalence of CVD risk factors among women. However, some studies do not report the actual percentage pertaining to the women studied and just presents the total percentage of both genders or male population only. Some studies were mixing adult and children within their included studies, hence some reported low prevalence. Moreover, some studies do not cover all the six members of the GCC countries, with some systematic reviews that present data from only two to three countries in the GCC region.

### Policy implications

This review indicated high levels of modifiable risk factors among women. Gender inequality damages the physical and mental health of millions of women across the globe. A continuous rising prevalence of lifestyle-related diseases increases the need for gender equality throughout the GCC countries, especially for Saudi Arabian women, to empower them in regards to their role in the society, their decision-making and more involvement in health care. Obesity is the major risk factor of CVDs in GCC countries and linked too many other NCDs. Women in GCC countries are facing a major struggle in challenging physical inactivity, which results in one of the highest obesity rates globally. Al-Bahilani and Mabry reported the legislations and policies issued by the GCC in regards behavioral risk of NCD, where most of them were related to tobacco control. However, in regards to the prevention of NCDs, only six policies have been addressed by the GCC’s ministries of health [68]. In 2012–2013, the GCC Secretary General, implemented short and long-term action plans to tackle NCDs, where short-term actions included “incentives and disincentives (such as taxes on tobacco), regulations (for example, limiting the availability of unhealthy food in schools), and clinical interventions (for instance, screening the population for risk factors)” [10].

Introducing a more active lifestyle by expanding the field of physical education through the GCC region and sports competitiveness among women is highly recommended. It is important to present a more elementary approach in measuring obesity levels by reporting central obesity with the combination of BMI, waist circumference and waist/hip ratio to obtain more accurate results. There is a high requirement for diet control and awareness in regards to total daily calorie intake. Although food labeling was introduced by the GCC customs union, the labeling requirements are basic and do not require regulations regarding the nutrition content of processed foods, such as sodium content and trans-fat [68]. Additionally, the direct and indirect costs of care and treatment of patients suffering from these diseases are significant and will become more burdensome as the price of oil has declined, and is likely to remain at lower levels due to the increased global supply. The data suggest that applying preventative measures for diabetes and CVD would potentially save 54% of the direct costs and 31% of the total cost of treatment. This results in not only a significant savings, but improved quality of life for the patients [10] and magnifies why the healthcare sector needs to focus more on preventable measures, such as motivating society to adopt healthy lifestyles. Implementing the health belief model and understanding health-related behavior among the female population in the GCC countries in regards to CVD and its risk factors would help in understanding why women are not adopting a healthier lifestyle.

## Conclusion

The high prevalence of lifestyle-related diseases among women population in GCC is a ticking time bomb and is reaching alarming levels, and require a fundamental social, cutural and political changes. These findings highlight the need for comprehensive work among the GCC to strengthen the regulatory framework to reduce and control the prevalence of these factors.

## Abbreviations

AHA: American heart association
AMSTAR: Assessment of multiple systematic review tool
ATP III: Adult treatment panel III
BMI: Body mass index
CVD: Cardiovascular disease
DALYs: Disability-adjusted life years
GCC: Gulf cooperation council
HBM: Health belief model
HTN: Hypertension
IDF: International diabetes federation
KSA: Kingdom of Saudi Arabia
MetS: Metabolic syndrome
NCEP: National cholesterol education program
UAE: United Arab Emirates
WHO: World health organization

## Footnotes

1 NCEP ATP III definition, metabolic syndrome is present if three or more of the following five criteria are met: waist circumference over 40 in. (men) or 35 in. (women), blood pressure over 130/85 mmHg, fasting triglyceride level over 150 mg/dl, fasting high-density lipoprotein (HDL) cholesterol level less than 40 mg/dl (men) or 50 mg/dl (women) and fasting blood sugar over 100 mg/dl.

2 IDF definition includes the same general criteria as the other definition; it requires that obesity, but not necessarily insulin resistance, be present.
